# A Scheme to Smooth Aggregated Traffic from Sensors with Periodic Reports

**DOI:** 10.3390/s17030503

**Published:** 2017-03-03

**Authors:** Sungmin Oh, Ju Wook Jang

**Affiliations:** Department of Electronics Engineering, Sogang University, Seoul 04107, Korea; dangkuk@sogang.ac.kr

**Keywords:** Internet of Things (IoT), wireless IoT sensor, offset, scheduling, traffic aggregation

## Abstract

The possibility of smoothing aggregated traffic from sensors with varying reporting periods and frame sizes to be carried on an access link is investigated. A straightforward optimization would take O(*p^n^*) time, whereas our heuristic scheme takes O(*np*) time where *n*, *p* denote the number of sensors and size of periods, respectively. Our heuristic scheme performs local optimization sensor by sensor, starting with the smallest to largest periods. This is based on an observation that sensors with large offsets have more choices in offsets to avoid traffic peaks than the sensors with smaller periods. A MATLAB simulation shows that our scheme excels the known scheme by M. Grenier et al. in a similar situation (aggregating periodic traffic in a controller area network) for almost all possible permutations. The performance of our scheme is very close to the straightforward optimization, which compares all possible permutations. We expect that our scheme would greatly contribute in smoothing the traffic from an ever-increasing number of IoT sensors to the gateway, reducing the burden on the access link to the Internet.

## 1. Introduction

The Internet of Things (IoT) is large scale by nature. This is not only manifested by the large number of connected devices but also by the huge volume of traffic that must be accommodated [1]. With the exponential growth of IoT devices [2,3], IoT networks will have to face the growth of traffic with the increasing amount of data exchanged between IoT sensors and servers [4]. For instance, in smart cities and smart buildings, IoT sensors with periodic transmission are used on a large scale [5,6].

**Figure 1:**
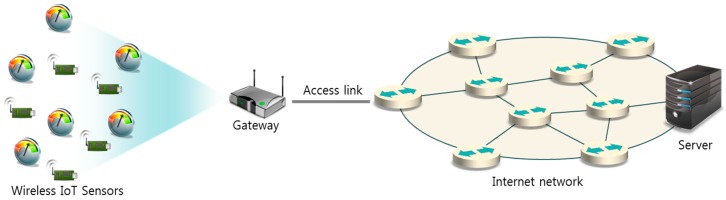
Wireless Internet of Things (IoT) Sensor Network model.

When periodic transmissions from a large number of wireless sensors with different periods and frame sizes are aggregated in the gateway to be carried on the access link as in Figure 1, the instant aggregated traffic can be bursty and far exceeds the average of the aggregated traffic [7]. To avoid congestion during possible bursty intervals, access link bandwidth should be much higher than needed for non-bursty traffic with the same average. The primary motivation for our work is to make the instant aggregated traffic as close to the average as possible by adjusting the offsets for individual sensors, thus reducing the access-link bandwidth needed to avoid instant congestion.

**Figure 2:**
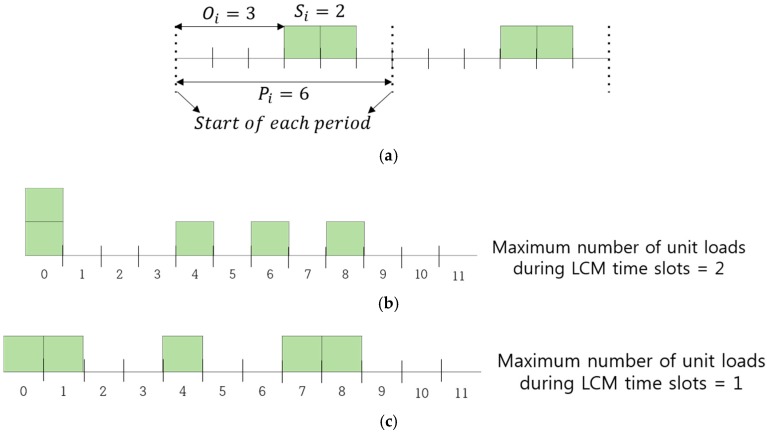
An illustrative example of *P_i_*, *S_i_*, *O_i_* and aggregation of unit traffic loads. (**a**) An illustrative example of *P_i_*, *S_i_*, *O_i_*; (**b**) aggregation of (*P*_1_ = 4, *S*_1_ = 1, *O*_1_ = 0) and (*P*_2_ = 6, *S*_2_ = 1, *O*_2_ = 0); (**c**) aggregation of (*P*_1_ = 4, *S*_1_ = 1, *O*_1_ = 0) and (*P*_2_ = 6, *S*_2_ = 1, *O*_2_ = 1).

Figure 2b,c in Section 3, Problem Definition, show two different aggregation scenarios for two sensors with periods 4 and 6. The average of the aggregated traffic during 12 (least common multiple (LCM) of 4 and 6) timeslots is the same for both scenarios, whereas the maximum of instant aggregated traffic load is 2 for (b) and 1 for (c). In Figure 2c, the offset for sensor 2 is changed from 0 to 1 (*O*_2_ = 1). Our objective is to arrange *O_i_* (*i* = 0, 1,…, *n* − 1) for *n* sensors in such a way as to minimize the maximum of the instant number of aggregated unit traffic loads during the *L* timeslots, where *L* is the LCM of *P*_0_, *P*_1_,…, *P_n−_*_1_, as the unit loads from *n* sensors are aggregated.

The efficiency of resource allocation and quality of service (QoS) that IP networks provide depends critically on effective traffic management [8]. Although a few studies have been performed, they mainly focus on traffic shaping [9] or traffic policing [10] by controlling the outbound gateway [11,12].

Traffic shaping, also known as packet shaping, is a network-management technique that delays certain types of packets to optimize overall network performance [13]. For example, Bell Canada, (Montreal, Canada) revealed that it throttles traffic from Peer to Peer (P2P) file-sharing applications in its broadband access networks to 256 Kbps per flow [14].

Traffic shaping is also applied to the aggregate traffic produced by multiple network flows. For instance, Comcast handles congestion in its access network by throttling users who consume a large portion of their provisioned access bandwidth over a 5-min time window [14,15].

Because these approaches focus on controlling the throughput, the gateway must monitor the traffic continuously, and this can be burdensome for the gateway. Additionally, this approach can introduce delay due to queuing, particularly deep queues [13].

Traffic policing allows us to control the maximum rate of traffic transmitted or received on an interface. Traffic policing is often configured on interfaces at the edge of a network to limit traffic in or out of the network. In most traffic-policing configurations, traffic that falls within the rate parameters is transmitted, whereas traffic that exceeds the parameters is dropped or transmitted with a different priority [16]. This approach drops excess packets (when configured), throttling Transmission Control Protocol (TCP) window sizes and reducing the overall output rate of affected traffic streams. Overly aggressive burst sizes may lead to excess packet drops and throttle the overall output rate [10].

Another method is to change the quality of the transmitted data in real time [17]. However, though suitable for voice- or video-data transmission, this method is not appropriate for sensor-data transmission.

The solution we propose is to distribute the periodic traffic from different sensors as evenly as possible to the access link in time and thus minimize the maximum of the instant traffic load on the access link. This can be achieved by scheduling sensors with offsets [18]. Precisely, the first instance of a stream of periodic frames is released with a delay, called the offset, with regard to a reference point, which is the first time at which the sensor is ready to transmit. Subsequent frames of the streams are then sent periodically, with the first transmission as the time origin. M. Grenier et al. proposed a scheme that schedules messages with offsets in a controller area network (CAN) to enhance CAN network performance [19]. The offset of each stream is chosen such that the release of its first frame is as far as possible from the other frames already scheduled. Similarly, we assume that if IoT sensors periodically transmit frames with optimal permutation of offsets, this can minimize the maximum of the instant traffic.

Goossens [20] has shown the problem of choosing the optimal permutation of offsets to have a complexity that grows exponentially with the periods of the tasks, and there is no known optimal solution that can be used in practical cases. Thus, in [20], only a few distinct values for the periods are allowed. A straightforward optimization would take O(*p^n^*) time, where *n*, *p* denote the number of sensors and size of periods, respectively.

In this paper, we propose a heuristic scheme that takes O(*np*) time. Our heuristic scheme performs local optimization sensor by sensor, starting with smallest to the largest periods. This is based on an observation that sensors with large offsets have more choices in offsets to avoid traffic peaks than sensors with smaller periods. A MATLAB (R2015b, MathWorks, Natick, MA, USA) simulation shows that our scheme excels the known scheme by M. Grenier et al. in a similar situation (aggregating periodic traffic in a CAN) for almost all possible permutations. The performance of our scheme is very close to the straightforward optimization, which compares all possible permutations. We expect our scheme will greatly contribute in smoothing the traffic from the ever-increasing number of IoT sensors to the gateway, reducing the burden on the access link to the Internet.

The rest of our paper is organized as follows: a wireless IoT sensor network model is provided in Section 2. The problem definition and proposed scheme are described in Section 3 and Section 4, respectively. The performance evaluation and time complexity of the proposed scheme are provided in Section 5 and Section 6, respectively, and Section 7 concludes the paper.

## 2. Wireless IoT Sensor Network Model

We model a wireless sensor network, as shown in Figure 1. A number of wireless sensors are connected to the gateway, and all traffic from the sensors is aggregated by the gateway to be carried on the access link to the Internet. The gateway aggregates sensor data, which is carried on the access link to the Internet. Table 1 summarizes the notations and variables used in this paper.

We assume a network in which sensors are characterized by the tuple sensor i = (*O_i_*, *P_i_*, *S_i_*). We further assume that we can control all sensors’ bandwidth evenly. The sensors’ offset exists in intervals [0, *P_i_* − *S_i_*]. Transmission is periodic, and thus, all sensors *i* (*i* = 0, 1,…, *n* − 1) transmit frames repeatedly at times *O_i_* + k*P_i_ (*k* is a non-negative integer).

## 3. Problem Definition

The *n* sensors, which transmit frames periodically, are connected to a gateway. Each sensor *i* is characterized by 3-tuple (*P_i_*, *S_i_*, *O_i_*), (*i* = 0, 1,…, *n* − 1), where *P_i_*, *S_i_*, and *O_i_* denote the period, frame size, and offset from the start of the period, respectively. Figure 2a illustrates how a 3-tuple (*P_i_*, *S_i_*, *O_i_*) is used. Sensor *i* generates a packet of size 2 (*S_i_* = 2) at the offset of 3 (*O_i_* = 3) from the start of each period of length 6 (*P_i_* = 6). We quantize the traffic from sensor *i* to be carried on the access link in such a way that transmission of a frame of *S_i_* unit loads occupies *S_i_* successive timeslots, contributing one unit load to each timeslot. We further assume that we may change *O_i_* (a non-negative integer) as long as 0 ≤ *O_i_* ≤ *P_i_* − *S_i_*.

All traffic from the *n* sensors is aggregated by the gateway to be carried on an access link to the Internet. Figure 2b,c illustrates how unit loads from sensor 1 and sensor 2 are aggregated. Because the same pattern of aggregation is repeated every LCM of *P*_1_ and *P*_2_, we only show 12 timeslots, which correspond to the LCM of 4 and 6. Figure 2b shows the aggregation of unit loads from sensors 1 (*P*_1_ = 4, *S*_1_ = 1, *O*_1_ = 0) and 2 (*P*_2_ = 6, *S*_2_ = 1, *O*_2_ = 0). Note that two unit loads are to be carried in timeslot 0, which implies in timeslot 0 that the access link needs two times the bandwidth needed in timeslots 4, 6, or 8. The maximum of instant aggregated traffic among 12 (LCM of *P*_1_ and *P*_2_) timeslots is 2. For this aggregation scenario, we need to assign enough bandwidth for the access link to accommodate the two unit loads to avoid congestion. Now, consider another aggregation scenario described in Figure 2c in which the offset of sensor 2 is changed to 1 (*O*_2_ = 1). Note that the maximum of instant aggregated traffic among 12 timeslots is now reduced to 1, needing half the bandwidth needed in Figure 2b. This illustrates how we reduce the bandwidth needed for an access link to carry aggregated traffic from sensors simply by coordinating their individual offsets.

Our objective is to arrange *O_i_* (*i* = 0, 1,…, *n* − 1) for *n* sensors in such a way to minimize the maximum of the instant number of aggregated unit traffic loads during the *L* timeslots where *L* is the LCM of *P*_0_, *P*_1_,…, *P_n−_*_1_, as the unit traffic loads from *n* sensors are aggregated. Because *O_i_* may have *P_i_* − *S_i_* + 1 possible choices, a straightforward optimization should compare all $\prod_{i=0}^{n-1}$ (*P_i_ − S_i_* + 1) cases. We will show our efficient heuristic algorithm, which compares only $\sum_{i-0}^{n-1}$ (*P_i_* − *S_i_* + 1) cases, in Section 4.

The following is a more formal definition of our problem.

Let B[*j*] (*j* = 0, 1,…, *L* − 1) denote the number of unit traffic loads in time slot *j* on the access link. *L* is the LCM of *P*_0_, *P*_1_,…, *P_n−_*_1_. If frames of sensor *i* with (*P_i_*, *S_i_*, *O_i_*) are carried on the access line, then B[*j*] (*j* = 0, 1,…, *L* − 1) is updated as follows. Note that $\frac{L}{P_{i}}$ frames of size *S_i_* are carried
for *k* = 0 to $\frac{L}{P_{i}}$ − 1,for *q* = 0 to *S_i_* − 1,B[*O_i_* + k*P_i_ + *q*] = B[*O_i_* + k*P_i_ + *q*] + 1.

Our objective is to find *O_i_* (*i* = 0, 1,…, *n* − 1), which minimizes the max(B) (the largest elements of the array B) after the following is performed.

Initially, B[*j*] = 0 (*j* = 0, 1,…, *L* − 1)
for *i* = 0 to *n* − 1 // for all *n* sensors,for *k* = 0 to $\frac{L}{P_{i}}$ − 1 // $\frac{L}{P_{i}}\;\mathrm{repetition}\;\mathrm{of}$
*P_i_*,for *q* = 0 to *S_i_* − 1 // frame of size *S_i_*,B[*O_i_* + k*P_i_ + *q*] = B[*O_i_* + k*P_i_ + *q*] + 1.

## 4. Proposed Scheme

We use the notations in Table 1 to describe our scheme. Sensor *i* is characterized by a 3-tuple (*P_i_*, *S_i_*, *O_i_*), (*i* = 0, 1,…, *n* − 1) where *P_i_*, *S_i_*, and *O_i_* denote the period, frame size, and offset from the start of the period, respectively. Without a loss of generality, we assume that *P_i_* ≤ *P_i+_*_1_ for *i* = 0, 1,…, *n* − 2.

### 4.1. Description of the Proposed Algorithm

The following is a brief description of our scheme.

(1) Sort *n* sensors in such a way that *P_i_* ≤ *P_i+_*_1_ for *i* = 0, 1,…, *n* − 2
(2) B[*j*], TEMP[*j*] (*j* = 0, 1,…, *L* − 1) ∈ *Z* (non-negative integer)
(3) for *j* = 0 to *L* − 1
B[*j*] = 0; TEMP[*j*] = 0; // Initializationmax(TEMP): the largest elements of the array TEMPstd(TEMP): $\sqrt{\frac{1}{L}\sum_{j=0}^{L-1}{\left(\mathrm{TEMP}\left[j\right]-\frac{1}{L}\sum_{j=0}^{L-1}\mathrm{TEMP}\left[j\right]\right)}^{2}}$
(4) for *i* = 0 to *n* − 1 // for all *n* sensors with an increasing order of period
  find *t* ∈ [0, *P_i_* − *S_i_*], which minimizes the max(TEMP) and std(TEMP) after the following operation
for *k* = 0 to $\frac{L}{P_{i}}$ − 1 // $\frac{L}{P_{i}}\;\mathrm{repetition}\;\mathrm{of}$
*P_i_*for *q* = 0 to *S_i_* − 1 // frame of size *S_i_*
  TEMP[t + k*P_i_ + q] = B[t + k*P_i_ + q] + 1; // incremental traffic by sensor *i* with offset *t*
  return *t*
*O_i_* = *t*;for *k* = 0 to $\frac{L}{P_{i}}$ − 1 // $\frac{L}{P_{i}}\;\mathrm{repetition}\;\mathrm{of}$
*P_i_*for *q* = 0 to *S_i_* − 1 // frame of size *S_i_*
  B[O_i_ + k*P_i_ + q] = B[O_i_ + k*P_i_ + q] + 1; // update B with traffic from sensor *i* with offset *O_i_*
  end for *i*.

In Algorithm 1 we now provide a pseudocode of our scheme using the notations in Table 1.

**Algorithm 1** The proposed algorithm
    max(TEMP): the largest elements of the array TEMP
    std(TEMP): $\sqrt{\frac{1}{L}\sum_{j=0}^{L-1}{\left(\mathrm{TEMP}\left[j\right]-\frac{1}{L}\sum_{j=0}^{L-1}\mathrm{TEMP}\left[j\right]\right)}^{2}}.$
    **circshift**(ADD_ONE, t): Shift right the array ADD_ONE by *t* positions1.**begin main**2. Sort *n* sensors in such a way that *P_i_* ≤ *P_i+1_* for *i* = 0, 1,…, *n* − 23. *L* ← (Global variable) the LCM of *P*_0_, *P*_1,_
*,..., P_n_*4. B[*j*] ← (Global variable) one-dimensional array, the length of which is *L* and initialized to 0 (*j* = 0, 1,…, *L* − 1)5. **for**
*i* = 0 to *n* – 1 // find best offset for sensor *i*, starting with the smallest to the largest period6.  *O_i_* ← **find_best_offset**(*S_i_, P_i_*)7.  B[0, 1,..., *L* − 1] ← **ADD_BLOCK**(*O_i_, P_i_, S_i_*); // update B with the traffic from sensor *i* with offset *O_i_*8. **end** for *i*9.**end main**

10.**find_best_offset**(*S_i_, P_i_*)11. **begin find_best_offset**12.  ADD_ONE[*j*] ← one-dimensional array, the length of which is *L* and initialized to 0 (*j* = 0, 1,…, *L* − 1)13.  TEMP[*j*] ← one-dimensional array, the length of which is *L* and initialized to 0 (*j* = 0, 1,…, *L* − 1)14. **for**
*k* = 0 to *L/P_i_* – 1 // $\frac{L}{P_{i}}\;\mathrm{repetition}\;\mathrm{of}$
*P_i_*

15.  **for**
*q* = 0 to *S_i_* – 1 // frame of size *S_i_*16.    ADD_ONE[k*P_i_+q] ← 1 // start with offset 017.  **end** for *q*18. **end** for *k*19. **for**
*j* = 0 to *L* − 120.   TEMP[*j*] ← B[*j*] + ADD_ONE[*j*] // incremental traffic with offset 021. **end** for *j*22.  Min_max ← **max**(TEMP)23.  Min_std ← **std**(TEMP)24.  offset ← 0 // start with offset = 025. **for**
*t* = 1 to *P_i_-S_i_*26.   ADD_ONE[0, 1,..., *L* − 1] ← **circshift**(ADD_ONE, 1) // This function circularly shifts the elements inarray ADD_ONE right by 1 position

27.  **for**
*j* = 0 to *L* − 128.    TEMP[*j*] ← B[*j*] + ADD_ONE[*j*]29.  **end** for *j*30.   temp_max ← **max**(TEMP)31.   temp_std ← **std**(TEMP)32.  **if** temp_max ≤ Min_max && temp_std < Min_std // max(TEMP) and std(TEMP) considered together33.   **then**34.    Min_max ← temp_max; Min_std ← temp_std; offset ← *t*;35.  **end**
**if**36.  **end** for *t*37.  **return** offset38. **end find_best_offset**

39.**ADD_BLOCK**(*O_i_, P_i_, S_i_*)40. **begin** ADD_BLOCK41. BLOCK[*j*] ← one-dimensional array, the length of which is *L* and is initialized to 0 (*j* = 0, 1,…, *L* − 1)42.  **for**
*k* = 0 to *L/P_i_* – 1 // $\frac{L}{P_{i}}\;\mathrm{repetition}\;\mathrm{of}$
*P_i_*43.   **for**
*q* = 0 to *S_i_* – 1 // frame of size *S_i_*44.    BLOCK[*O_i_*+k*P_i_+q] ← 1 // the incremental traffic loads from sensor *i* with offset *O_i_*45.  **end** for *q*46.  **end** for *k*47.  **for**
*j* = 0 to *L* − 148.   B[*j*] ← B[*j*] + BLOCK[*j*]; // update B with the traffic from sensor *i* with offset *O_i_*49.  **end** for *j*50.  **return** B[0, 1,..., *L* − 1]51. **end**
**ADD_BLOCK**

Array B[*j*] (*j* = 0, 1,…, *L* − 1) in Algorithm 1 is a one-dimensional array, the length of which is *L*. Let B[*j*] (*j* = 0, 1,…, *L* − 1) denote the number of unit traffic loads in time slot *j* on the access link. *L* is the LCM of *P*_0_, *P*_1_,…, *P_n−_*_1_. The following procedure is repeated for *i* = 0, 1,…, *n* − 1.

**Figure 3:**
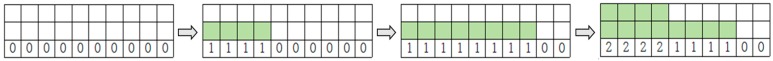
Gaps may result if our search for offset stops for only the first minimum of the max(TEMP).

The offset is determined by find_best_offset subroutine. First, the array ADD_ONE[*j*] is declared and all elements are initialized to 0 (*j* = 0, 1,…, *L* − 1). TEMP[*j*] and ADD_ONE[*j*] are used to try each offset. ADD_ONE[*j*] represents the incremental traffic with each trial offset, and TEMP[*j*] represents the incremented traffic with the trial offset (refer to TEMP[*j*] = B[*j*] + ADD_ONE[*j*], (*j* = 0, 1,…, *L* − 1)). In other words, ADD_ONE[*j*] will be circularly shifted right with increasing *t* (*t* ∈ Z and 0 ≤ *t* ≤ *P_i_ − S_i_*). The *t* that minimizes the maximum value and the standard deviation of TEMP[*j*] (*j* = 0, 1,…, *L* − 1) is returned as *O_i_*. The primary goal of the proposed algorithm is to minimize the maximum value of B[*j*]. However, if our search for the offset stops at the first minimum of max(TEMP), there may be a chance that some gaps will not be filled, causing the minimum of the final max(B) to increase in the next or a later round. Consider Figure 3, in which *P_i_* = 10 and *S_i_* = 4. The minimum of the max(TEMP) is two for *O_i_* = 0, 1, 2, 3, 4, 5, and 6. If we choose a number other than 6 for *O_i_*, we will end up with the gap in time slot 9 and/or 8. Based on this reasoning, we choose an offset that not only minimizes max(TEMP) but also std(TEMP). Our algorithm uses max(TEMP) and std(TEMP) together (as shown in the find_best_offset subroutine in our pseudocode) to fill the possible gaps in *P_i_* − (*P_i_* mod *S_i_*), *P_i_* − (*P_i_* mod *S_i_*) + 1, …, (*P_i_* − 1)th timeslot as best as possible.

The standard deviation std(TEMP) is calculated by the following formula:

(1) $$\sqrt{\frac{1}{L}\overset{L-1}{\underset{j=0}{\sum }}{\left(TEMP\left[j\right]-\frac{1}{L}\overset{L-1}{\underset{j=0}{\sum }}TEMP\left[j\right]\right)}^{2}}.$$

**Figure 4:**
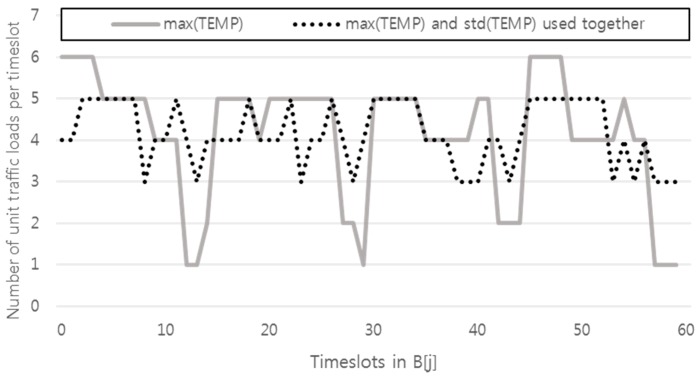
The number of unit traffic loads in timeslots depending on how offsets are chosen. For *i* = 0 to 9, *P_i_* = 4, and *S_i_* = 15. For *i* = 10 to 19, *P_i_* = 3, and *S_i_* = 20.

Figure 4 illustrates our point. The solid line in Figure 4 shows the variation of unit traffic loads per timeslot when max(TEMP) is only used, whereas the dotted line shows the variation when max(TEMP) and std(TEMP) are used together. Note that the variation of the dotted line is far smoother than that of the solid line. The final max(B) for the dotted line is 5, whereas the final max(B) for the solid line is 6.

**Figure 5:**
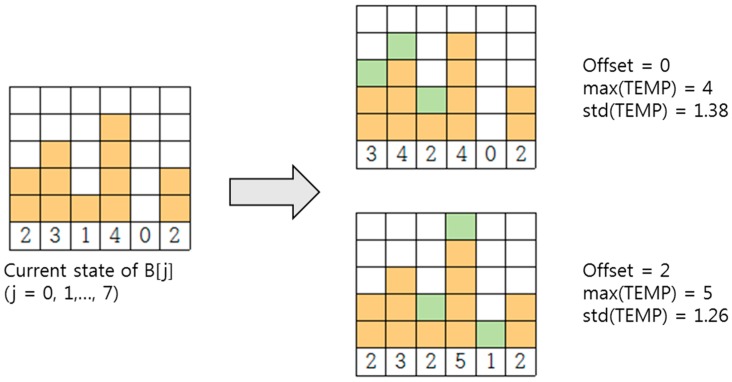
Lower std(TEMP) does not mean lower max(TEMP).

However, std(TEMP) alone is not enough to choose the best offset to minimize the final max(B). Figure 5 illustrates this point. For an offset of 0, max(TEMP) is 4 and std(TEMP) is 1.38, whereas for an offset of 2, max(TEMP) is 5 and std(TEMP) is 1.26. Lower std(TEMP) does not mean lower max(TEMP). If we choose the offset with the smallest std(TEMP), we would choose an offset of 2, which results in a max(TEMP) of 5 (bottom of the right side in Figure 5). However, max(TEMP) for an offset of 0 is 4, which is lower than 5 (top of the right side in Figure 5).

Based on this observation, we compare max(TEMP) and std(TEMP) when choosing the best offset.

After the offset is determined, we update array B with the incremental traffic loads from sensor *i*. The subroutine ADD_BLOCK creates a one-dimensional array BLOCK[*j*], *j* = 0 to *L* − 1, which is initialized to all 0s. The incremental traffic loads from sensor *i* are loaded into BLOCK[*j*], *j* = 0 to *L* − 1 as follows: for *k* = 0 to $\frac{L}{P_{i}}$ − 1 and for *q* = 0 to *S_i_* − 1, BLOCK[*O_i_* + k*P_i_ + *q*] is set to 1. Then, the summation B[*j*] = B[*j*] + BLOCK[*j*] is performed for the update by update B with the traffic from sensor *i*.

### 4.2. Comparison against a Previous Work by M. Grenier et al.

Assigning offsets for “traffic shaping” is a problem that has been addressed in [20,21] concerning the preemptive scheduling of tasks. M. Grenier et al.’s [19] work is the closest to our scheme to the best of our knowledge. It adjusts offsets for messages in such a way that spreads the messages over time as much as possible on the CAN (a shared bus for the transmission of messages in a car) to minimize worst case response time (WCRT).

Automotive message sets have certain specific characteristics (a small number of different periods, etc.) shared by the periodic frames from IoT sensors. However, the aim of our scheme is to minimize the demand for instant bandwidth, reducing the burden on the access link that connects the gateway (collecting traffic from many periodic frames from IoT sensors) to the Internet.

Here, we implement M. Grenier et al.’s scheme for comparison against our scheme. A brief description of their scheme is provided below for convenience:

We assume that the streams are sorted by increasing value of their period, i.e., *k* < *h* implies *T_k_* ≤ *T_h_*. The algorithm sets iteratively the offsets of streams from *f*_1_ to *f_n_*. Let us consider that the stream under analysis is *f_k_*.

Set the offset for *f_k_* to maximize the distance between its first release *f_k_*_,1_, and the release right before and right after *f_k_*_,1_. Concretely,

(a) Look for the smallest load in the interval [0, *T_k_*];
(b) Look for one of the longest least-loaded intervals in [0, *T_k_*] for which ties are broken arbitrarily. The first (resp. last) possible release time of the interval is noted by *B_k_* (resp. *E_k_*);
(c) Set the offset *O_k_* in the middle of the selected interval; the corresponding possible release time is *r_k_*;
(d) Update the release array *R* to store the frames of *f_k_* released in the interval [0, T_max_]:
∀*i* ∈ N and r_k_ + $i\cdot \frac{T_{k}}{g}$ ≤ $\frac{T_{max}}{g}$,do R$\left[r_{k}+i\cdot \frac{T_{k}}{g}\right]$ = R$\left[r_{k}+i\cdot \frac{T_{k}}{g}\right]$ ∪ $f_{k,i+1},$(g: granularity of offsets).

### 4.3. An Illustrative Example of the Proposed Algorithm

**Figure 6:**
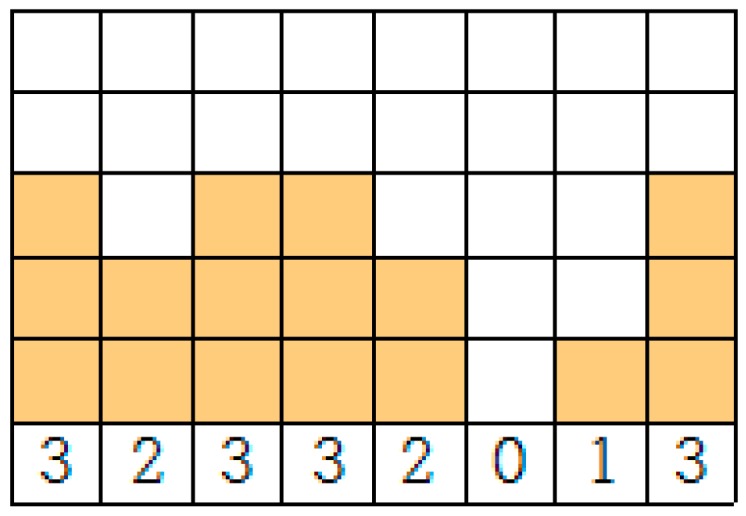
An example of the current state of B[*j*] (*j* = 0, 1,…, 7).

**Figure 7:**
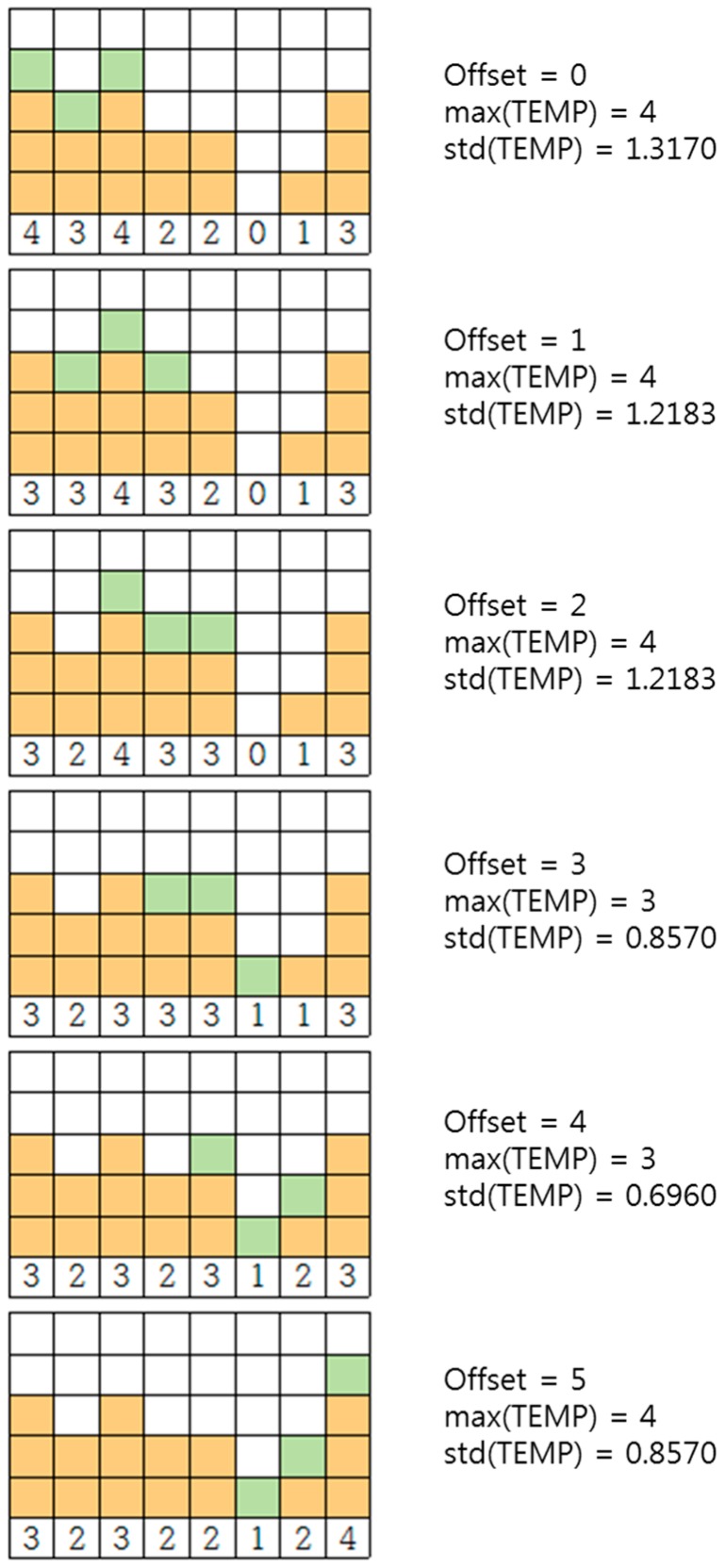
An offset determination process.

We provide an illustrative example of our scheme in Figure 6. In this example, we assume that the current traffic load is represented as an array B[*j*] (*j* = 0, 1,…, 7) in Figure 6. A block represents a unit traffic load. For example, B[0], B[1], and B[2] have three, two, and three units of traffic loads, respectively. We show the process of determining the offset for a sensor with *P_i_* = 8 and *S_i_* = 3, as in Figure 7.

Figure 7 shows the trial of all possible offset values (because *P_i_* − *S_i_* = 5, we have 0, 1, 2, 3, 4, and 5). The green blocks represent incremental traffic loads with specific offset values. The max(TEMP) and std(TEMP) are shown on the right along with the corresponding offset values. Offset 4 is chosen because it results in the lowest std(TEMP) as well as minimum max(B).

## 5. Performance Evaluation

We present the simulation results of the proposed scheme. Furthermore, we compare the simulation results against M. Grenier et al. and a base implementation of random offset assignment. Thus, we implement three different simulations: (1) random offset (base implementation); (2) M. Grenier et al.; and (3) the proposed scheme.

When sensors periodically transmit frames with random offsets (a base implementation), the max(B) may differ with regard to individual instances of the simulation. We performed 100 iterations to obtain the confidence interval and the average for max(B). The confidence interval is obtained by using the max(B)’s mean denoted by $\bar{\mu }$ and the standard deviation denoted by σ. Let *B_r_* denote the array B in iteration *r*, *r* = 0, 1, …, *R* − 1, where *R* is the number of iterations (*R* = 100 in our simulation). The $\bar{\mu }$ and σ are obtained as in Equations (2) and (3):

(2) $$\bar{\mu }=\frac{\sum_{r=1}^{R}\max\left(B_{r}\right)}{R},$$

(3) $$\sigma =\frac{\sum_{r=1}^{R}{\left(\max\left(B_{r}\right)-\frac{\sum_{r=1}^{R}\max\left(B_{r}\right)}{R}\right)}^{2}}{R-1}.$$

The confidence interval with a 95% confidence level can be obtained using normal distribution, as in Equation (4) [22]:

(4) $$\bar{\mu }-1.645\frac{\sigma }{\sqrt{R}}\leq \mu \leq \bar{\mu }+1.645\frac{\sigma }{\sqrt{R}}.$$

The simulation is performed with varying values of *n* (number of sensors), *P_i_* (transmission period of sensors), and *S_i_* (size of frames for sensors).

[*n*: variable, *P_i_* and *S_i_*: fixed] We compare the performances of three schemes: random offset (base), M. Grenier et al. and the proposed scheme. In the case of random offsets, which exhibit different results on each iteration, the mean and confidence intervals for max(B) are shown.

**Figure 8:**
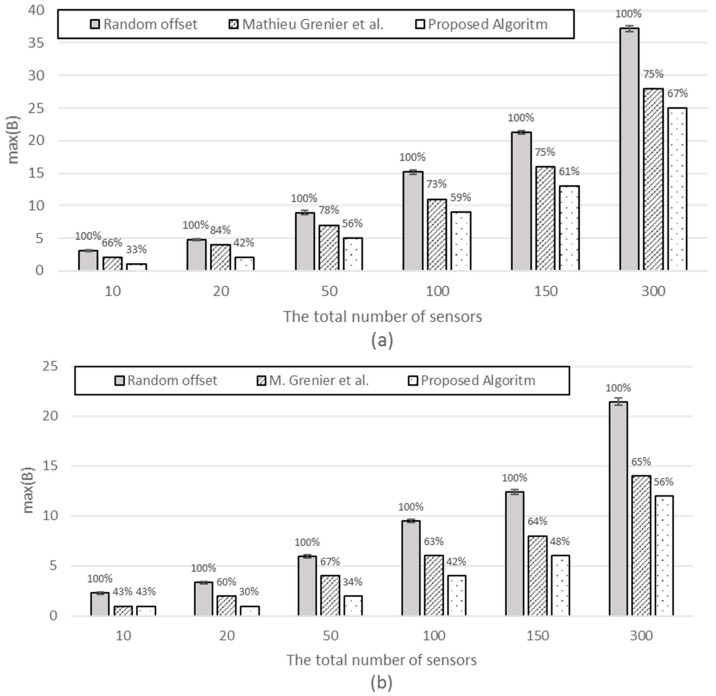
The max(B) vs. number of sensors with (**a**) *P_i_* = 60 and *S_i_* = 5; (**b**) *P_i_* = 100 and *S_i_* = 4.

Figure 8 shows that the performance of the three schemes with the max(B) for the random offsets (base implementation) is set to 100%. Figure 8 shows that our scheme results in the minimum max(B) and is followed by M. Grenier et al. and then the random offset (from 33%, 66%, and 100% for *n* = 10 to 56%, 65%, and 100% for *n* = 300). As the number of sensors increases, the relative differences tend to diminish due to statistical multiplexing. Our scheme excels over other schemes in that the minimum max(B) implies the lowest burden on the access link.

**Figure 9:**
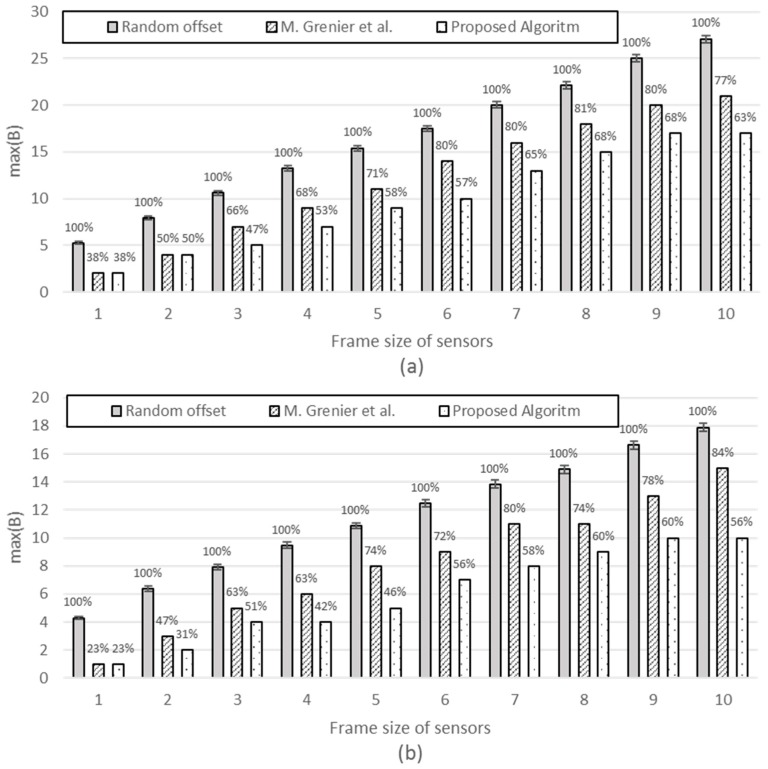
max(B) with (**a**) *n* = 100, and for all *i*, *P_i_* = 60; (**b**) *n* = 100, and for all *i*, *P_i_* = 100.

[*n*, *P_i_*: fixed, *S_i_* : variable] The simulation is performed with an increasing frame size 1 to 10. Figure 9 compares the three schemes, with the proposed scheme exhibiting the minimum. When the frame size is as small as 1 or 2, our scheme shows similar or smaller max(B) compared with M. Grenier et al. because, for small frames, it does not help very much in reducing max(B) to try all the possible offsets within its period to find the best timeslot to fit the frame. However, as the frame size increases, the efficiency of the proposed scheme excels that of M. Grenier et al. This is more evident for greater periods (the difference is greater for Figure 9b *P_i_* = 100 than Figure 9a *P_i_* = 60).

**Figure 10:**
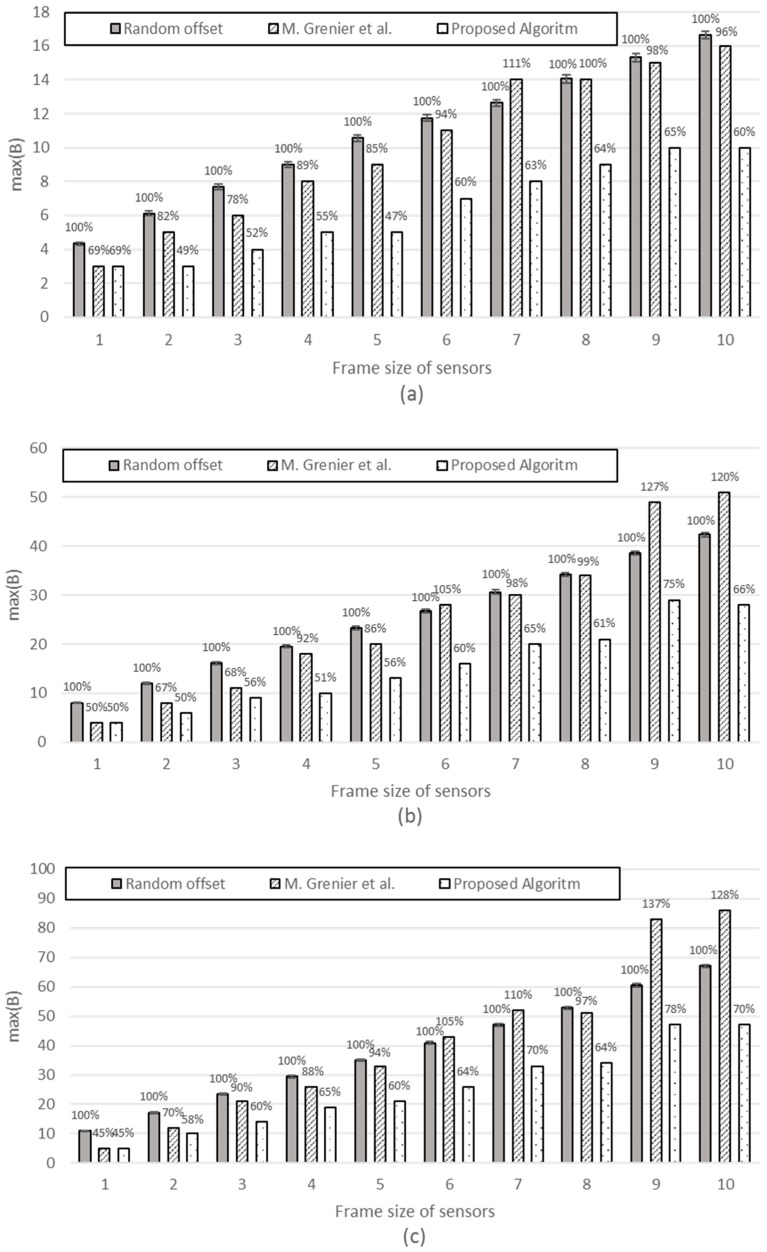

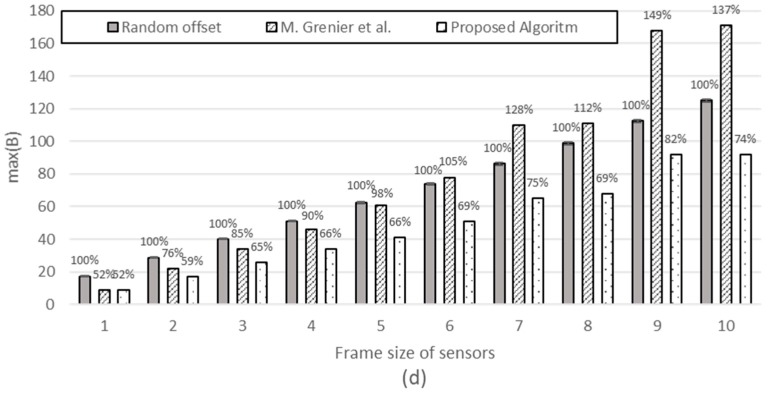
The max(B). For *i* = 0 to $\frac{1}{3}$*n* − 1, *P_i_* = 25. For *i* = $\frac{1}{3}$*n* to $\frac{2}{3}$*n* − 1, *P_i_* = 40. For *i* = $\frac{2}{3}$*n* to n − 1, *P_i_* = 70 (**a**) *n* = 30; (**b**) *n* = 90; (**c**) *n* = 150; (**d**) *n* = 300.

[*n*,: fixed, *P_i_*, *S_i_*: variable] Figure 10 compares the three schemes with each third of the sensors having different *P_i_*, with the frame size increasing from 1 to 10.

It is interesting to note that M. Grenier et al. performs better than the random offset (base scheme) with smaller frames; however, it performs worse than the random offset scheme as the frame size increases. This becomes more evident with *n* increasing from 30 to 300. This implies that M. Grenier et al. is only applicable for small-sized frames. The proposed schemes exhibit stable gain against random offsets and M. Grenier et al. regardless of the frame size or number of sensors.

**Figure 11:**
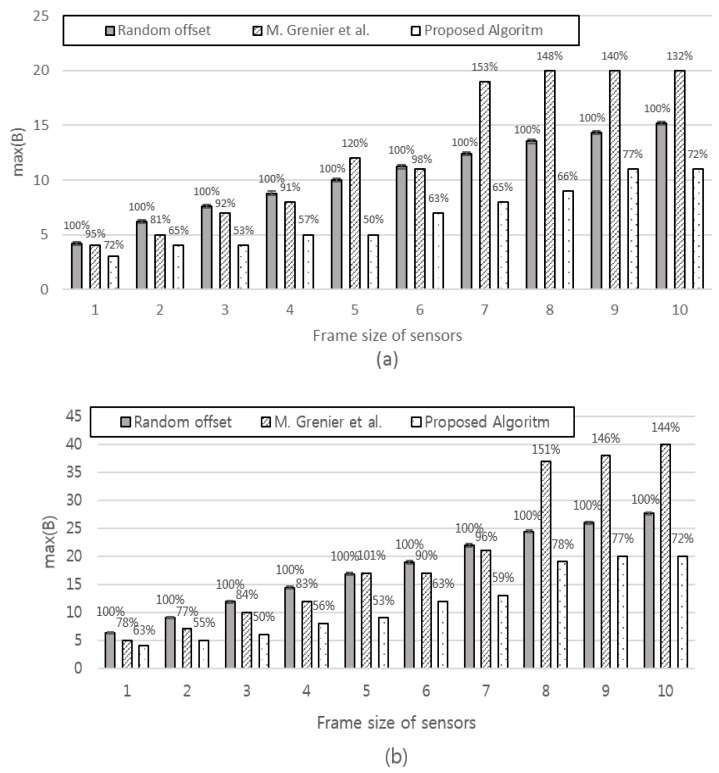

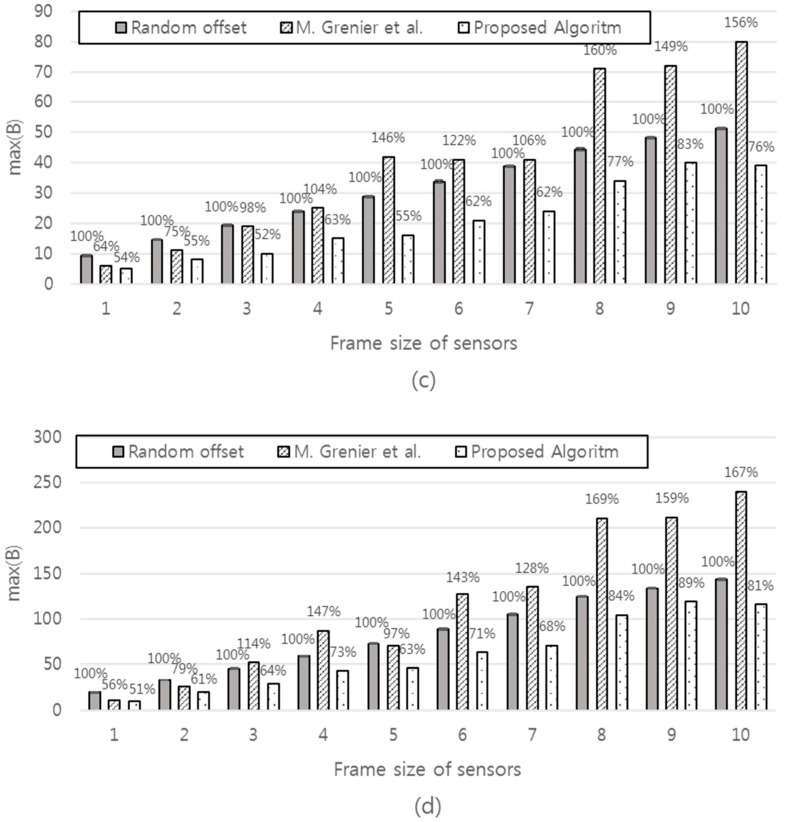
The max(B). For *i* = 0 to $\frac{1}{5}$*n* − 1, *P_i_* = 15. For *i* = $\frac{1}{5}$*n* to $\frac{2}{5}$*n* − 1, *P_i_* = 25. For *i* = $\frac{2}{5}$*n* to $\frac{3}{5}$*n* − 1, *P_i_* = 40. For *i* = $\frac{3}{5}$*n* to $\frac{4}{5}$*n* − 1, *P_i_* = 70. For *i* = $\frac{4}{5}$*n* to *n* − 1, *P_i_* = 200 (**a**) *n* = 30; (**b**) *n* = 90; (**c**) *n* = 150; (**d**) *n* = 300.

Figure 11 compares the three schemes with each fifth of the sensors having different *P_i_* with frame sizes, increasing from 1 to 10. We find a similar tendency as in Figure 10.

**Figure 12:**
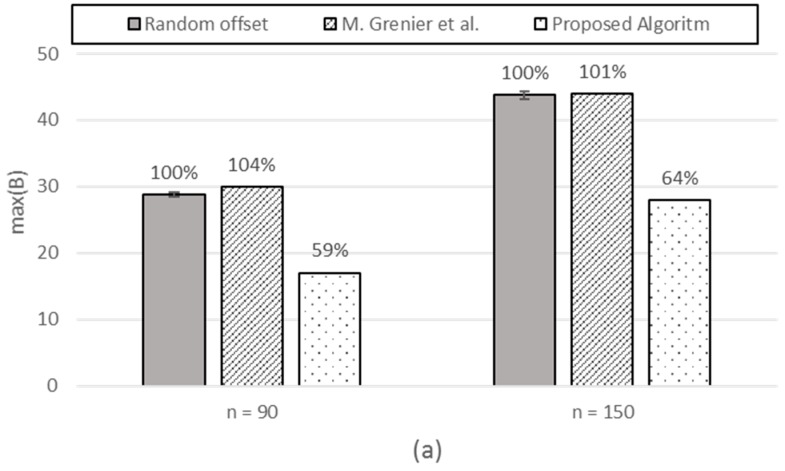

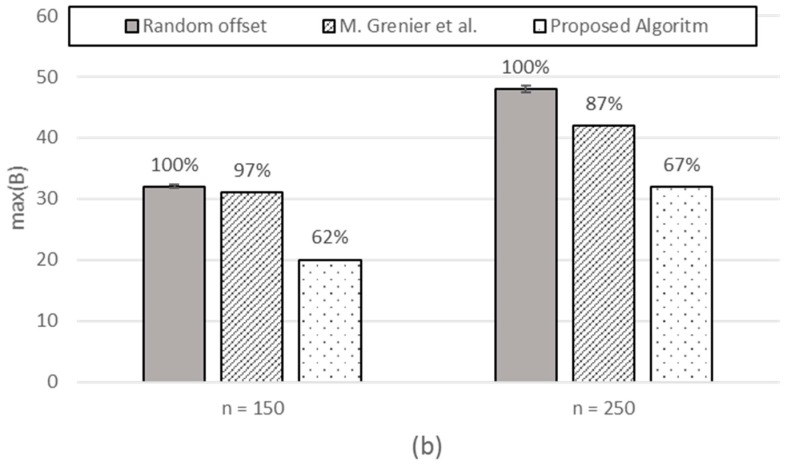
The max(B). (**a**) For *i* = 0 to $\frac{1}{3}$*n* − 1, *P_i_* = 25 and *S_i_* = 5. For *i* = $\frac{1}{3}$*n* to $\frac{2}{3}$*n* − 1, *P_i_* = 40 and *S_i_* = 7. For *i* = $\frac{2}{3}$*n* to n − 1, *P_i_* = 70 and *S_i_* = 10; (**b**) For *i* = 0 to $\frac{1}{5}$*n* − 1, *P_i_* = 15 and *S_i_* = 2. For *i* = $\frac{1}{5}$*n* to $\frac{2}{5}$*n* − 1, *P_i_* = 25 and *S_i_* = 3. For *i* = $\frac{2}{5}$*n* to $\frac{3}{5}$*n* − 1, *P_i_* = 40 and *S_i_* = 5. For *i* = $\frac{3}{5}$*n* to $\frac{4}{5}$*n* − 1, *P_i_* = 70 and *S_i_* = 7. For *i* = $\frac{4}{5}$*n* to *n* − 1, *P_i_* = 200, *S_i_* = 10.

[*n*: fixed, *P_i_*, *S_i_*: variable (*P_i_*, *S_i_* changes together as a pair)] Figure 12 shows that the proposed scheme shows robust gain against both schemes, whereas the performance gain for a random offset or M. Grenier et al. against each other depends on the mixture of periods and frame sizes.

**Figure 13:**
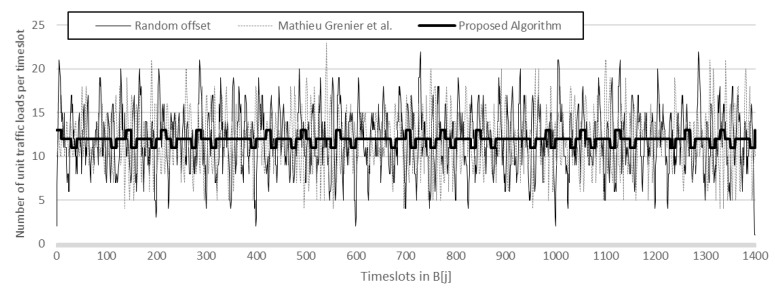
The number of unit traffic loads in B[*j*] for *j* = 0, 1,…, 1399 (for sensor *i* =0 to 29, *P_i_* = 25; for *i* = 30 to 59, *P_i_* = 40; for *i* = 60 to 89, *P_i_* = 70 and for all *i*, *S_i_* = 5).

Figure 13 shows the number of unit traffic loads in B[*j*] for *j* = 0, 1,…, 1399. There are 1400 timeslots (B[*j*] for *j* = 0, 1,…, 1399) because the LCM of 25, 40, and 70 is 1400. The proposed scheme in a heavy solid line exhibits far smoother traffic compared with a random offset (light solid line) and M. Grenier et al. (dotted line).

Based on the above simulation, we conclude that the efficiency of our scheme is very robust in smoothing traffic on the access link, which carries aggregated traffic from sensors with periodic transmission (possibly different periods and/or frame sizes or a various mixture).

## 6. Time Complexity of the Proposed Scheme

We consider the time complexity of determining the optimal permutation of offsets for all of the sensors. Sensor *i* has *P_i_* − *S_i_* + 1 choices of offsets because the offset can be chosen from 0, 1, 2, …, *P_i_* − *S_i_*. If we choose any offset greater than *P_i_* − *S_i_*, say *P_i_* − *S_i_* + *x*, *x* > 0, then the frame of size *S_i_* needs to be transmitted beyond the current period of size *P_i_* (*P_i_* − *S_i_* + *x* + *S_i_* > *P_i_* for *x* > 0). Here, we assume that a frame of size *S_i_* consists of *S_i_* unit traffic loads and takes *S_i_* timeslots. Because the offset can be chosen independently for each sensor, we have $\prod_{i=0}^{n-1}$ (*P_i_* − *S_i_* + 1) permutations. Straightforward optimization would evaluate all of these permutations to obtain the best performance in smoothing the aggregated traffic. Its time complexity can be represented by O(*p^n^*) time, for which *n*, *p* denote the number of sensors and size of periods, respectively.

In contrast, our heuristic scheme evaluates only $\sum_{i-0}^{n-1}$ (*P_i_* − *S_i_* + 1) permutations. The reason for this reduced complexity is that we do not evaluate all possible permutations of $\prod_{i=0}^{n-1}$ (*P_i_* − *S_i_* + 1). We rather optimize sensor by sensor, starting with the sensor with the smallest period. We evaluate *P*_0_* − S*_0_ + 1 choices of offsets for sensor 0. For each possible offset, we can evaluate the incremental unit traffic loads contributed by sensor 0 and choose the best offset *O*_0_ that best smoothes the resulting traffic (i.e., minimizes the maximum of the instant number of aggregated unit traffic loads during the *L* timeslots in which *L* is the LCM of *P*_0_, *P*_1_,…, *P_n−_*_1_ as the unit loads from *n* sensors are aggregated). This is repeated for sensors 1, 2, …, in sequence. Because each sensor *i* needs *P_i_* − *S_i_* + 1 evaluations, our scheme evaluates $\sum_{i-0}^{n-1}$ (*P_i_* − *S_i_* + 1) permutations in total, which can be represented by the time complexity of O(*np*).

Our heuristic scheme greatly saves computation time. For example, if there are five sensors with a *P*_1_ = 15, *P*_2_ = 25, *P*_3_ = 25, *P*_4_ = 40, *P*_5_ = 70 and *S*_1_ = 2, *S*_2_ = 3, *S*_3_ = 3, *S*_4_ = 4, *S*_5_ = 5, a straightforward optimization should compare 14,723,280 permutations, whereas our scheme compares only 158 cases. On our computer simulation with a 3.4-GHz quad-core CPU, a straightforward optimization took 3024 s, whereas our scheme took less than a second. M. Grenier et al.’s scheme takes a similar time as our scheme. The random offset has no computation time because it allows sensors to individually determine their respective offsets. Simply, the straightforward optimization takes O(*p^n^*) time, whereas our scheme and M. Grenier et al. take O(*np*) time, for which *n*, *p* denote the number of sensors and size of periods, respectively.

The difference in max(B) of our scheme against the straightforward optimization was zero in the above particular simulation. We expect the difference would remain zero or be very small for most cases. Our scheme determines offsets starting with sensors with the smallest to the largest periods. On each iteration with a sensor, we thoroughly investigate all possible offsets to find the offset that minimizes the current max(B) and std(B). Note that the sensors with smaller periods have fewer choices in offsets and that the sensors with larger periods have more choices in offsets. Thus, sensors with large offsets have more choices in offsets to avoid traffic peaks than those with smaller periods. We did not consider many cases of straightforward optimization because a few instances of straightforward optimization would take a formidable amount of computation time.

## 7. Conclusions

We have investigated the possibility of smoothing aggregated traffic from sensors with varying reporting periods and frame sizes via a gateway to be carried on an access link by adjusting the offsets of the periodic transmission from individual sensors. A straightforward optimization would consider all possible permutations of offset values, i.e., $\prod_{i=0}^{n-1}$ (*P_i_* − *S_i_* + 1) permutations, for which *P_i_* and *S_i_* denote the period and frame size, respectively. Its time complexity can be represented by O(*p^n^*) time, for which *n*, *p* denote the number of sensors and size of periods, respectively.

Our heuristic scheme takes only $\sum_{i-0}^{n-1}$ (*P_i_* − *S_i_* + 1) permutations, which can be represented by the time complexity of O(*np*). We perform local optimization sensor by sensor in ascending order of periods, starting with the sensor 0 with the smallest period to the sensor *n* − 1 with the largest period. We evaluate *P_i_* − *S_i_* + 1 choices of offsets for sensor *i*. For all choices, we evaluate the incremental unit traffic loads contributed by sensor *i* and choose the offset *O_i_* that best smoothes the resulting traffic (i.e., minimizes the maximum of the instant number of aggregated unit traffic loads during *L* timeslots, in which *L* is the LCM of *P*_0_, *P*_1_,…, *P_n−_*_1_ as the unit loads from *n* sensors are aggregated). This is performed for *i* = 0 to *n* − 1 in sequence. Because sensor *i* needs *P_i_* − *S_i_* + 1 evaluations, our scheme evaluates $\sum_{i-0}^{n-1}$ (*P_i_* − *S_i_* + 1) permutations, which can be represented by the time complexity of O(*np*).

M. Grenier et al. is closest to our scheme. It adjusts offsets for messages in such a way that spreads the messages of different periods over time as much as possible on the CAN to minimize WCRT. It is similar to our scheme in that it performs local optimization with increasing value of periods. The difference from our scheme is that it looks for the longest least-loaded interval and sets the offset to the middle of the selected interval. The time complexity of the scheme can be represented by the same O(*np*) as ours because the task of finding the longest least-loaded interval is O(*p*).

The advantage of our scheme over M. Grenier et al.’s scheme lies in the performance of the traffic smoothing (i.e., maximum of the instant number of aggregated unit traffic loads). The maximum in our scheme is as low as half of their scheme, depending on the number of sensors, periods, and frame sizes, but never higher than their scheme because our scheme also includes their scheme in our evaluation. An extensive MATLAB simulation shows that our scheme excels over the scheme by M. Grenier et al. for almost all possible permutations in the number of sensors, periods, and frame sizes. Especially, as the frame sizes increase, the performance gap tends to grow.

The performance of our scheme is very close to the straightforward optimization that compares all possible permutations. In our scheme, the computational overhead is greatly reduced from exponential O(*p^n^*) time to linear O(*np*) time. We expect our scheme would greatly contribute in smoothing the traffic from the ever-increasing number of IoT sensors to the gateway, reducing the burden on the access link to the Internet.

The proposed scheme is naturally heuristic because it does not consider all possible permutations on offsets for all of the sensors. However, it has been shown to be very efficient in smoothing traffic on the access link. The local optimization of each sensor’s traffic is performed starting with sensors with the smallest periods to those with the largest periods. The time complexity of our scheme is greatly reduced compared to brute-force optimization with all possible permutations.

## Figures and Tables

**Table 1:** Notations and variables.

Notations/Variables	Meaning
*i*	Sensor number. Each sensor has a number *i* (*i* = 0, 1,…, *n* − 1)
*P_i_*	Period of sensor *i*. Time interval from the start of the current frame transmission to the start of the next frame transmission (in the number of unit timeslots)
*S_i_*	Frame size of sensor *i*. (in the number of unit traffic loads)
*O_i_*	Offset of sensor *I* (0 ≤ *O_i_* ≤ *P_i_* − *S_i_*)
*L*	Least Common Mupltiple (LCM) of *P_0_*, *P_1_*,…, *P_n_*_−1_
B[*j*] (*j* = 0, 1,…, *L* − *1*)	One-dimensional array, the length of which is *L*. B[*j*] represents the number of unit traffic loads in timeslot *j*.
ADD_ONE[*j*] (*j* = 0, 1,…, *L* − 1)	One-dimensional array, the length of which is *L*. ADD_ONE[*j*] represents an incremental unit traffic loads in timeslot *j*, thus either 0 or 1.
TEMP[*j*] (*j* = 0, 1,…, *L* − 1)	One-dimensional array, the length of which is *L*. TEMP[*j*] represents the sum of B[*j*] and ADD_ONE[*j*].
Min_max	The smallest max(TEMP) found so far (the function max returns the largest elements of an array)
Min_std	The smallest std(TEMP) found so far (the function std returns the population standard deviation of an array)
temp_max	Current max(TEMP)
temp_std	Current std(TEMP)
BLOCK[*j*] (*j* = 0, 1,…, *L* − 1)	One-dimensional array, the incremental traffic loads from sensor *i* are loaded into this array
